# 肺癌术后静脉血栓栓塞症相关危险因素分析：单中心研究

**DOI:** 10.3779/j.issn.1009-3419.2018.10.04

**Published:** 2018-10-20

**Authors:** 松平 崔, 辉 李, 博 田, 春凤 宋, 滨 胡

**Affiliations:** 100020 北京，首都医科大学附属北京朝阳医院胸外科 Department of Thoracic Surgery, Beijing Chaoyang Hospital, Capital Medical University, Beijing 100020, China

**Keywords:** 静脉血栓栓塞症, 下肢深静脉血栓形成, 肺癌, 胸外科手术, Venous thromboembolism, Deep venous thrombosis of lower extremity, Lung neoplasms, Thoracic surgery

## Abstract

**背景与目的:**

肺癌术后静脉血栓栓塞症（venous thromboembolism, VTE）的发生率并非少见，本研究旨在分析肺癌术后VTE相关危险因素，为进一步防治VTE提供临床依据。

**方法:**

本研究为单中心研究。选择2016年7月-2017年12月在首都医科大学附属北京朝阳医院胸外科接受手术治疗的肺癌患者，除术前常规检查外，所有患者均在手术前后行下肢多普勒超声以明确有无新发下肢深静脉血栓（deep venous thrombosis, DVT）。患者术前术后均未接受预防性抗凝治疗。按照术后是否发生VTE将患者分为VTE组和对照组，比较两组间的基线资料、手术相关资料（手术类型、术式等）、肿瘤病理相关资料（病理分型、血管浸润、病理分期等）。

**结果:**

按照纳入标准，本研究共对339例接受肺癌手术的患者进行了分析，其中男性166例，女性173例，年龄范围23岁-86岁。术后共39例发生VTE，发生率为11.5%。比较两组年龄、性别、体质量指数（body mass index, BMI）、美国麻醉医师协会（American Society of Anesthesiologists, ASA）、吸烟状况、基础疾病等，除年龄有显著性差异外，其他指标均无显著性差异；比较两组术前血常规、血生化、凝血、肿瘤标记物、肺功能、下肢静脉超声等，术前癌胚抗原（carcinoembryonic antigen, CEA）水平、术前D-二聚体水平、肺功能水平及下肢肌间静脉扩张比率两组有显著性差异，其他指标均无显著性差异；比较两组手术时长、手术方式、出血量、病理类型、病理分期、血管侵犯等，手术时长、手术方式（开胸*vs*胸腔镜）、出血量等因素有统计学差异，其他指标均无显著性差异。单因素分析结果显示：VTE组在年龄、术前CEA水平、术前D-二聚体水平、肺功能水平、下肢肌间静脉扩张比率、开胸手术比率、手术时长、出血量均与对照组有显著差异（*P* < 0.05）。而病理分期、病理类型等因素与VTE发生无显著相关性。多因素*Logistics*回归分析显示，第1秒用力呼气量（forced expiratory volume in one second, FEV_1_）、手术方式、下肢肌间静脉扩张是肺癌术后合并VTE的独立危险因素（*P* < 0.05）。

**结论:**

本研究结果提示患者FEV_1_、手术方式以及下肢肌间静脉扩张是肺癌术后合并VTE的独立危险因素。

## Abstract (en)

**Background and objective:**

The Previous study has indicated that the incidence of venous thromboembolism (VTE) after lung cancer surgery is not uncommon. The aim of this study is to analyze the risk factors of postoperative VTE in lung cancer patients and provide a clinical basis for further prevention and treatment of VTE.

**Methods:**

This study was a single-center study. From July 2016 to December 2017, all patients with lung cancer who underwent surgery in our department were enrolled into this study. Except routine preoperative examinations, lower extremity Doppler ultrasound was performed in all patients before and after surgery to determine whether there was any newly developed deep venous thrombosis (DVT). Patients did not receive any prophylactic anticoagulant therapy before and after surgery. Patients were then divided into VTE group and control group according to whether VTE occurred after operation. Baseline data, surgical related data (surgery type, surgical procedure, *etc*.) and tumor pathological data (pathological type, vascular infiltration, pathological staging, *etc*.) were compared between the two groups.

**Results:**

According to the inclusion criteria, a total of 339 patients undergoing lung cancer surgery were analyzed. There were 166 males and 173 females with an age range of 23-86 years. A total of 39 patients developed VTE after surgery, the incidence rate of postoperative VTE was 11.5%. Comparing the age, gender, body mass index (BMI), American Society of Anesthesiologists (ASA), smoking status, underlying diseases, *etc*, there were no significant differences in other indicators except for significant differences in age; comparison between preoperative blood routine, blood biochemistry, coagulation, tumor markers, lung function, lower extremity venous ultrasound, preoperative carcinoembryonic antigen (CEA) levels, preoperative D-dimer levels, there were significant differences in lung function and lower extremity intermuscular vein expansion ratio. There were no significant differences in other indexes between the two groups. The duration of surgery, surgical procedure, bleeding volume, pathological type, pathological stage, vascular invasion, were compared between the two groups. There were statistical differences in surgical methods (thoracic *vs* thoracoscopic) and bleeding volume. There were no significant differences in other indicators. Univariate analysis showed that age, preoperative CEA level, preoperative D-dimer level, poor pulmonary function, lower extremity intermuscular vein dilation ratio, thoracotomy rate, length of surgery, and amount of bleeding were significantly risk factors (*P* < 0.05). There were no significant correlations between pathological stage and pathological type and VTE. Multivariate logistic regression analysis showed that forced expiratory volume in one second (FEV_1_), surgical approach, and lower extremity intermuscular vein dilatation were independent risk factors for postoperative VTE in patients with lung cancer (*P* < 0.05).

**Conclusion:**

The results of this study suggest that FEV_1_, surgical procedures, and lower extremity intermuscular vein dilation are independent risk factors for postoperative VTE in patients with lung cancer.

静脉血栓栓塞症（venous thromboembolism, VTE）是胸外科术后的严重并发症之一，一旦发生不但延长了患者的住院时间，占用了大量的医疗资源，对患者的预后也造成了严重的影响。本课题组先期的研究已经证实了肺癌术后VTE的发生率是16.4%^[1]^。但是肺癌术后发生VTE究竟与哪些因素相关，我国目前尚无相关研究。本研究的目的是通过分析肺癌术后发生VTE患者的临床资料，试图寻找高危因素，为今后临床实践和进一步研究提供证据。

## 1 资料与方法

### 1.1 研究对象

本研究为单中心研究，以2016年7月-2017年12月于我院胸外科接受肺癌手术治疗的病例为研究对象。纳入标准：①原发性肺癌患者；②接受手术治疗；③手术前后均行下肢静脉超声检查。排除标准：①肺转移癌；②术前下肢超声明确有深静脉血栓（deep vein thrombosis, DVT）；③患者术后未行下肢多普勒超声检查；④有其他血液系统疾病；⑤术前、术后因其他疾病应用抗凝治疗。

### 1.2 资料收集

采集一般资料：所有研究对象资料采用统一的资料收集表格，其中包括患者的基本信息（年龄、性别、合并疾病、吸烟史、体质量指数等）、实验室检查（白细胞计数、中性比、血小板计数、D-二聚体、肿瘤标记物等）、肺功能检查[第1秒用力呼气量（forced expiratory volume in one second, FEV_1_）、最大通气量（maximal voluntary ventilation, MVV）等]、手术的相关信息（手术方式、切除范围、手术时长、出血量等）、肿瘤病理资料（肿瘤大小、分类、分化程度、血管浸润、脉管癌栓等）。

VTE相关检查：所有患者行Caprini风险分层，评分≤4分为VTE低危、5分-8分为中危和≥9分为高危。入组患者手术前术后均行下肢多普勒超声以明确有无DVT，如存在以下几项情况之一，还需行CT肺动脉造影（computed tomography pulmonary angiography, CTPA）除外有无新发肺栓塞（pulmonary embolism, PE）：①出现肺血栓栓塞症（pulmonary thromboembolism, PTE）典型症状（胸痛、咯血或无法解释的低氧血症和呼吸困难）；②Caprini评分≥9分；③术后新发的DVT。

### 1.3 诊断依据及标准

肺癌的诊断以肿瘤病理学结果为依据，肺癌的分期参照2016年国际抗癌联盟（Union for International Cancer Control, UICC）推荐的第八版肺癌TNM分期标准；深静脉血栓经彩色多普勒超声检查诊断，PTE经CT肺动脉造影诊断。术后VTE事件定义为术前明确无VTE的患者，术后经下肢静脉超声诊断为深静脉血栓形成（deep vein thrombosis, DVT）（包括肌间静脉血栓）或经CTPA诊断为PE。

### 1.4 统计学方法

呈正态分布计量资料用均数±标准差（Mean±SD）表示，采用*t*检验进行组间比较；不服从正态分布的计量资料用M（QR）表示，采用非参数检验进行组间比较；计数资料采用构成比和率表示，用χ^2^检验进行组间比较。将单因素分析结果中有统计学意义的变量纳入多因素*Logistic*回归分析。所有数据均应用SPSS 21.0数据软件包进行数据统计分析，以*P* < 0.05为差异有统计学意义。

## 2 结果

### 2.1 基线资料分析

2016年7月-2017年12月，共339例患者入组，其中术后诊断VTE 39例（VTE组），其中DVT 35例，PTE 1例，DVT合并PE 13例；未发生VTE 300例（对照组）。VTE组年龄明显大于对照组年龄（65.3±1.4 *vs* 59.4±0.6, *P*=0.01）；在性别、BMI、ASA评分、吸烟、合并症等方面，VTE组与对照组无统计学差异（表 1）。

**1:** 肺癌患者基线信息分析 Analysis of baseline information of lung cancer patients

	VTE (*n*=39)	Non-VTE (*n*=300)	*χ*^2^/*t*	*P*
Gender			1.112	0.292
Male	16	150		
Female	23	150		
Age (Mean±SD, yr)	65.3±1.4	59.4±0.6	-3.475	0.010
BMI (Mean±SD, kg/m^2^)	24.4±0.8	23.9±0.2	-0.513	0.608
ASA			3.001	0.083
1-2	32	276		
3-5	7	24		
Smoking			0.000	0.987
< 400	28	215		
≥400	11	85		
Hypertension			0.001	0.979
No	28	216		
Yes	11	84		
Coronary heart disease			0.106	0.745
No	36	285		
Yes	3	15		
Diabetes			1.020	0.313
No	37	264		
Yes	2	36		
VTE: venous thromboembolism; BMI: Body mass index.				

### 2.2 术前辅助检查资料分析

所有患者术前均行血常规、血生化、凝血七项、肿瘤标记物、肺功能、下肢静脉超声等检查。其中白细胞计数、中性比、红细胞计数、血红蛋白、红细胞压积、血小板计数在VTE组与对照组间均无统计学差异。血生化检查：术前血钠、总胆红素、白蛋白水平在VTE组与对照组间无统计学差异。肿瘤标记物检查：VTE组与对照组间除CEA水平有统计学差异（10.1±4.1 *vs* 7.4±2.0, *P*=0.014）外，ProGRP、AFP、SCC、NSE、CA125、CYFRA211水平均无统计学差异。VTE组术前D-二聚体水平高于对照组（0.548±0.13 *vs* 0.389±0.05），且具有统计学差异（*P*=0.001）。肺功能检查：VTE组FEV_1_、PEF、MVV、DLCOcSB均低于对照组，且具有统计学差异。下肢静脉超声示VTE组肌间静脉扩张比率高于对照组，且具有统计学差异（*P*=0.000）（表 2）。

**2:** 肺癌患者术前辅助检查信息分析 Analysis of preoperative auxiliary examination information in patients with lung cancer

	VTE (*n*=39)	Non-VTE (*n*=300)	*χ*^2^/*t*	*P*
Blood routine and biochemical examination				
White blood cell count	5.8±0.3	6.3±0.1	-1.252	0.211
Neutral ratio	60.5±1.7	60.5±0.6	0.031	0.975
Red blood cell count	4.4±0.1	4.5±0.0	-1.294	0.197
Hemoglobin	133.6±2.4	137.3±0.9	-0.918	0.358
Hematocrit	38.9±0.6	39.9±0.2	-1.098	0.272
PLT	245.8±13.0	239.2±3.7	-0.254	0.799
Preoperative blood sodium	141.4±0.3	141.8±0.1	-0.969	0.333
Preoperative total bilirubin	12.1±0.8	12.5±0.3	-0.430	0.667
Preoperative albumin	41.3±0.7	42.0±0.2	-1.116	0.264
Tumor marker				
ProGRP	35.9±2.0	42.8±4.3	-0.979	0.327
CEA	10.1±4.1	7.4±2.0	-2.461	0.014
AFP	2.9±0.3	3.0±0.2	-0.613	0.540
SCC	1.1±0.2	1.6±0.3	-0.774	0.439
NSE	16.3±0.8	15.4±0.3	-1.235	0.217
CA125	15.7±3.1	18.6±4.1	-1.938	0.053
CYFRA211	3.3±0.8	3.0±0.3	-0.415	0.678
Preoperative D-dimer	0.5±0.1	0.4±0.1	-3.270	0.001
Pulmonary function tests				
FEV_1_	2.1±0.1	2.6±0.0	-3.657	0.000
PEF	6.0±0.3	7.6±0.4	-3.387	0.001
MVV	87.0±3.8	105.5±1.8	-3.623	0.000
DLCOcSB	6.6±0.3	7.4±0.1	-2.264	0.024
Lower extremity venous ultrasound			14.028	0.000
Normal	19	235		
Abnormal	20	65		
FEV_1_: forced expiratory volume in one second; MVV: maximal voluntary ventilation; CEA:				

### 2.3 手术相关因素分析

本组包括左肺手术133例（39.2%），右肺手术206例（60.8%）；手术方式为开胸手术74例（21.8%），胸腔镜手术265例（78.2%）；手术切除部位为上叶165例（48.8%），中叶13例（3.8%），下叶76例（23.3%），复合肺叶85例（25.1%）。手术术式包括肺局部切除（楔形切除和肺段切除）47例（13.9%），肺叶切除（含复合肺叶切除）286例（84.3%），全肺切除6例（1.8%）。开胸手术患者的术后VTE发生率明显高于胸腔镜手术患者，统计学有显著差异（*P*=0.024）。VTE组手术时长、出血量均大于对照组，且有统计学差异（*P*=0.01, *P*=0.04）。VTE组住院时长较对照组长，但无统计学差异（*P*=0.466）（表 3）。

**3:** 手术相关因素分析 Analysis of surgical related factors

	VTE (*n*=39)	Non-VTE (*n*=300)	*χ*^2^/*t*	*P*
Surgical site			0.206	0.650
Left	14	119		
Right	25	181		
Surgical procedure			5.112	0.024
VATS	25	240		
Open thoracotomy	14	60		
Scope of surgery			0.612	0.736
Partial lung resection	4	43		
Lobectomy	34	252		
Compound lobectomy	1	5		
Length of surgery (Mean±SD, min)	197.6±9.2	172.8±3.0	-2.562	0.01
Bleeding (Mean±SD, mL)	302.6±79.7	173.2±9.5	-2.058	0.04
Length of hospital stay (Mean±SD, d)	14.1±0.9	13.0±0.3	-0.730	0.466

### 2.4 病理相关因素分析

本组术后病理结果显示为小细胞肺癌11例（3.2%），非小细胞肺癌328例（97.8%）；其中肺鳞癌63例（18.6%），肺腺癌260例（76.7%）。TNM分期：0期-Ⅱ期279例（85.1%），Ⅲ期-Ⅳ期49例（14.9%）。病理相关因素分析示：病理类型、血管浸润、脉管癌栓、TNM分期、病理分期在VTE组与对照组间均无统计学差异（表 4）。

**4:** 病理相关因素分析 Analysis of pathological factors

	VTE (*n*=39)	Non-VTE (*n*=300)	*χ*^2^/*t*	*P*
SCLC	0	11		
NSCLC	39	289	1.064	0.302
Adenocarcinoma	29	231		
Squamous cell carcinoma	10	53		
Vascular invasion			0.023	0.879
No	30	234		
Yes	9	66		
Vascular tumor thrombus			0.008	0.929
No	34	260		
Yes	5	40		
T stage			3.347	0.067
Tis+1	26	230		
2+3+4	13	59		
N stage			1.726	0.189
0+1	31	252		
2	8	37		
M stage	0	0		
Pathological staging			2.307	0.129
0+Ⅰ+Ⅱ	30	249		
Ⅲ+Ⅳ	9	40		
SCLC: small cell lung cancer; NSCLC: non-small cell lung cancer.				

### 2.5 多因素*Logistic*回归分析

将上述单因素分析中有统计学意义的因素（年龄、CEA、D-二聚体、肺功能、肌间静脉扩张、手术时长、手术方式、出血量是肺癌术后合并VTE的危险因素）纳入多因素*Logistic*回归分析，结果显示：下肢肌间静脉扩张、手术方式、FEV_1_水平是肺癌手术患者合并静脉血栓栓塞症的独立危险因素（*P* < 0.05）（见表 5）。

**5:** 肺癌手术患者合并静脉血栓栓塞的*Logistics*回归分析 *Logistic* regression analysis of venous thromboembolism in patients with lung cancer surgery

	B	*P*	OR	95%CI
CEA	0.727	0.118	2.1	0.8-5.1
D-dimer	0.515	0.277	1.7	0.7-4.2
FEV_1_	-0.902	0.014	0.4	0.2-0.8
Surgical procedure	0.841	0.038	2.3	1.0-5.1
Length of surgery	0.891	0.170	2.4	0.7-8.7
Bleeding	0.058	0.897	1.1	0.4-2.6
Lower extremity venous ultrasound	1.400	0.000	4.1	2.0-8.3

## 3 讨论

VTE是外科手术后常见并发症之一，也是影响患者预后的重要原因。肺癌患者发生VTE的机制十分复杂，涉及遗传性和获得性因素的相互关联，多种机制导致了患者血纤溶-抗凝系统的失衡，癌细胞及其代谢产物使得患者血液处于高凝状态。研究证实肺癌是发生VTE的高危因素^[2]^，在我们先前的研究中肺良性肿瘤术后VTE的发生率是7.5%，恶性肿瘤发病率是16.4%^[1]^，也进一步证实了该观点。为明确肺癌手术患者合并VTE的相关危险因素，我们开展了这项单中心研究。

本研究的患者基线资料单因素分析中，高龄是肺癌患者发生VTE的危险因素（*P*=0.01），性别、BMI、ASA、吸烟量、基础疾病与VTE的发生没有显著相关性。回顾文献发现，年龄与VTE发生的相关性有争议，部分研究认为高龄是肺癌合并VTE的高危因素^[3]^，另一部分则认为年龄的增大反而降低了VTE的发生率^[4]^，我们认为这可能与研究对象的选择相关，本研究以肺癌手术患者作为研究对象，首先部分高龄不耐受手术的患者未纳入研究，因而数据可能有所偏倚。近期一项研究显示，高血压等心血管疾病会增加VTE的发病率^[5]^，在本研究中未得到证实。

血清检验分析中，CEA和术前D-二聚体水平在VTE组与对照组间有统计学差异，而白细胞计数、中性比、红细胞计数、血红蛋白、红细胞压积、血小板计数、术前血钠、总胆红素、白蛋白水、ProGRP、AFP、SCC、NSE、CA125、CYFRA211水平无统计学差异。CEA水平与VTE相关性在既往文献中提及较少，但也有部分文献支持CEA水平的升高与PE的发生相关^[5]^，这与本研究相符；D-二聚体水平代表了患者术前的凝血状态^[6]^，其敏感性高而特异性较差，因此多用于VTE的辅助诊断与预后监测^[7]^。有部分研究认为白细胞计数的升高、中性比升高、血小板计数升高、血红蛋白水平降低会增加VTE的发生率^[8-10]^，白蛋白水平、术前血钠水平降低^[11, 12]^也会增加VTE发生的风险，但在本研究中VTE组与对照组未显示统计学差异。

在Caprini评分中，肺功能是重要的评分项目。在本研究中我们纳入了患者术前肺功能的相关资料，如FEV_1_、PEF、MVV、DLCOcSB等。在单因素分析中，VTE组肺功能相关数据均低于对照组，且具有统计学差异。考虑到肺功能与年龄有多重共线性，因而在多因素分析中我们人为地剔除了年龄，并将FEV_1_作为肺功能的代表性指标纳入多因素分析，结果显示FEV_1_水平是肺癌合并VTE的独立危险因素。我们推测这可能与缺氧造成的血管内皮损伤有关，但仍需更多的研究数据证实。在下肢静脉超声方面，我们把下肢肌间静脉扩张定义为下肢超声结果异常，未见明显异常则为正常。在单因素分析中，下肢超声结果异常与VTE有明确相关性，继而纳入多因素分析，结果显示下肢超声结果异常是肺癌合并VTE的独立危险因素。下肢肌间静脉通常指小腿肌间静脉丛与跖静脉丛、大腿内收静脉丛同属下肢深静脉丛，位于小腿腹侧肌群中，由于分支多，静脉管径细小，管壁薄，静脉瓣数量较少，血流速度缓慢，以及周围为无深筋膜等坚硬组织等原因，因而容易形成血栓。目前认为肌间静脉血栓是DVT的一种类型，通常起病隐匿，病变范围小，尤其是年轻患者常常临床症状较轻, 多被忽视。近期一项纳入1, 027例肿瘤患者的研究显示静脉曲张增大了VTE发生的概率^[13]^，这与我们的研究结果相符。

肺癌手术涉及重要血管的阻断，不可避免会造成局部动静脉的损伤，而血管内皮的损伤会使胶原和基底膜暴露，同时释放组织因子（tissue factor, TF），促进血小板的凝集，导致血栓形成；血管的阻断也会导致动静脉压力、血液流速以及凝血平衡的改变，促进血栓的形成；手术过程中的静卧以及术后的长时间卧床，可导致下肢肌肉泵回流作用减弱，下肢静脉扩张患者静脉血回流受阻，易发生血栓事件。在手术相关因素的分析中，手术方式（开胸*vs*胸腔镜）、手术时长、出血量在VTE组与对照组间有统计学差异，手术切除方式未见统计学差异，VTE组住院时间大于对照组，但无统计学差异。开胸手术相比于胸腔镜手术创伤大，术后恢复期长，术后疼痛导致卧床时间更长可能是VTE发生率增高的原因；手术长时间的制动以及麻醉药物的影响增加了VTE的发生率；出血量与VTE的相关性主要体现在凝血平衡的破坏以及特殊情况下输血对血流动力学的改变^[2]^。本研究中肺癌手术患者以早期肺腺癌为主，手术切除方式以肺叶切除为主，肺楔形切除、亚肺叶切除及全肺切除数量较少，未表现出与VTE发生的相关性。多项研究显示全肺切除是发生VTE的高危因素^[3]^，但也有文献显示手术切除范围与VTE的发生无关^[14]^。本组VTE组住院时长大于对照组，但无统计学差异。发生VTE的患者需要接受抗凝治疗，一定程度上会延长住院时间，尤其是发生PE的患者^[15, 16]^，同时也会造成更多的医疗费用以及护理费用^[17]^。

肿瘤细胞可以直接表达TF来激活凝血，也可以通过分泌IL-1、IL-6、肿瘤坏死因子等作用于血管内皮细胞、纤维母细胞、巨噬细胞，使之上调组织因子的表达、调控蛋白C途径、活化血小板，间接激活凝血，进而促进血栓形成。肿瘤病理资料分析中，病理类型、血管侵犯、脉管癌栓、病理分期等因素在VTE组与对照组间无统计学差异，但多项研究显示肺腺癌、肿瘤分化程度低、肿瘤晚期、N3是VTE的高危因素^[3, 18-22]^。我们考虑由于本研究纳入的是接受手术的肺癌患者，肿瘤分期较早，病理类型多为肺腺癌（76.7%），因而数据可能存在偏倚。

目前，胸外科肺癌术后患者预防性抗凝用药并不规范，且抗凝药的选择、用量、有效性、安全性有待评估，因而本研究未纳入抗凝相关因素的分析。本研究的研究对象为接受手术的肺癌患者，目的是明确其危险因素，相比于其他肺癌合并VTE的研究，结论有一定局限性，仍需更多临床数据的支持。

综上所述，本研究结果提示：年龄、CEA水平、D-二聚体水平、肺功能、肌间静脉扩张、手术方式、手术时长、出血量是肺癌术后VTE的危险因素，其中，FEV_1_、手术方式、下肢肌间静脉扩张是其独立危险因素。
